# Genome Sequence of Bacillus velezensis 5RB, an Overproducer of 2,3-Butanediol

**DOI:** 10.1128/MRA.01475-18

**Published:** 2019-01-03

**Authors:** Penka Petrova, Petya Velikova, Kaloyan Petrov

**Affiliations:** a Institute of Microbiology, Bulgarian Academy of Sciences, Sofia, Bulgaria; b Institute of Chemical Engineering, Bulgarian Academy of Sciences, Sofia, Bulgaria; University of Maryland School of Medicine

## Abstract

Bacillus velezensis 5RB is capable of producing large amounts of 2,3-butanediol. Whole-genome sequencing revealed that the strain contains one circular chromosome of 3.91 Mbp without plasmids. A large part of the genome is devoted to carbohydrate metabolism, encoding an abundance of enzymes participating in polysaccharide utilization pathways.

## Abstract (precis)

Bacillus velezensis 5RB is capable of producing large amounts of 2,3-butanediol. Whole-genome sequencing revealed that the strain contains one circular chromosome of 3.91 Mbp without plasmids.

## ANNOUNCEMENT

The organic chemical 2,3-butanediol (2,3-BD) is the starting reagent in chemical syntheses and an ingredient in foods and pharmaceuticals (1). Biotechnological approaches for 2,3-BD production have progressed over the past decade, turning 2,3-BD into a major product of mixed-acid fermentations (2, 3). Currently, the aims are to use nonpathogenic *Bacillus* strains (4) and convert renewable raw materials (5).

B. velezensis 5RB was isolated in the Veliko Tarnovo region of Bulgaria from lake sediment containing plant roots. Single colonies of the strain were grown in nutrient broth (Oxoid) at 30°C. Genomic DNA was extracted using a GeneJET genomic DNA purification kit (Thermo Fisher Scientific). The TruSeq DNA PCR-free kit was used for library construction; the sequencing was performed on an Illumina HiSeq 2500 instrument with FastQC quality control (Macrogen, Inc., South Korea). Quality-filtered data contained 43,639,513,900 total bases and 289,794,196 read counts. The assembly was done using SOAPdenovo2 software (6) yielding 26 contigs with a total length of 3,910,395 bp, 134.22× genome coverage, an *N*_50_ value of 394,584 bp, and a 46.5% G+C content. The NCBI Prokaryotic Genome Annotation Pipeline (7) detected 4,605 genes, 3,745 of them encoding proteins, 81 tRNAs, and 8 rRNAs.

Strain 5RB belongs to the Bacillus amyloliquefaciens operational group (8), with a 99% similarity with soy isolate B. velezensis YJ11-1-4 (GenBank accession number NZ_CP020874) (9). *In silico* DNA-DNA hybridization (DDH) (10) resulted in a DDH value of 90.20% with the *B. velezensis* FZB42 genome (CP000560) and a relatively lower DDH of 85.7% with that of the type strain NRRL B-41580 (LLZC00000000).

B. velezensis 5RB contains genes which are typical for plant-associated rhizobacterial genomes (11–13). The metabolic model of Rapid Annotations using Subsystems Technology (RAST) (default settings) (14) built by ModelSEED v2.3 predicted a 2,3-BD synthesis pathway engaging *ilvB*, *alsS*, and *ilvH* (encoding α-acetolactate synthase), *alsD* (α-acetolactate decarboxylase), and *bdhA* [(R,R)-2,3-butanediol dehydrogenase; EC 1.1.1.4]. The last enzyme was identical to the 2,3-butanediol dehydrogenase of B. amyloliquefaciens KHG19 (GenBank accession number CP007242) (15) but different from those of B. velezensis FZB42 and NRRL B-41580^T^, which may explain the overproduction of 2,3-BD by B. velezensis 5RB.

A large portion of the genome of B. velezensis 5RB is devoted to carbohydrate metabolism (225 genes). The following genes encode glycoside hydrolases: *amyE*, *malL*, *sacA*, *xynA*, *xynB*, *xynD*, *xynC*, and *eglS*. This rich enzyme spectrum enables the conversion of cellulose, hemicellulose, starch, and inulin and is promising for the use of B. velezensis 5RB to produce 2,3-BD in biotechnological processes for simultaneous saccharification and fermentation (SSF) of renewable plant substrates.

Secondary metabolite production was analyzed using the antiSMASH v4.2.0 tool (16). Seven complete genomic clusters encoding antimicrobials were observed. Three of them encode the synthesis of the polyketides macrolactin, bacillaene, and difficidin, and four of them encode the nonribosomal production of fengycin, bacillibactin, bacilysin, and the cyclic lipopeptide surfactin. The synthesis by B. velezensis 5RB of a number of substances with an antibiotic nature would allow its application in industrial microbial fermentations in nonsterile conditions.

### Data availability.

This whole-genome sequencing (WGS) project has been deposited at DDBJ/ENA/GenBank under the accession number QXJL00000000 (raw data are available under SRA numbers SRX5028064 and SRR8208868).
